# Early post-traumatic seizures are associated with valproic acid plasma concentrations and UGT1A6/CYP2C9 genetic polymorphisms in patients with severe traumatic brain injury

**DOI:** 10.1186/s13049-017-0382-0

**Published:** 2017-08-25

**Authors:** Yirui Sun, Jian Yu, Qiang Yuan, Xing Wu, Xuehai Wu, Jin Hu

**Affiliations:** Department of Neurosurgery, Huashan Hospital, Fudan University, 12 Wulumuqi Road, Shanghai, 200040 People’s Republic of China

**Keywords:** Traumatic brain injury, Valproic acid, Seizure, UGT1A6, CYP2C9, Genetic polymorphisms

## Abstract

**Background:**

Seizure is a common complication for severe traumatic brain injury (TBI). Valproic acid (VPA) is a first-line antiepileptic drug, though its metabolism is affected by genetic polymorphisms and varies between individuals. The aim of this study was to investigate such association and to explore its influence on the occurrence of early post-traumatic seizure.

**Methods:**

A prospective case control study was conducted from 2012 to 2016 recruiting adult patients with severe TBI. Electroencephalograph (EEG) monitoring was performed approximately 4 h for each patient from day 1 to day 7 after injury. If seizures were detected, EEG monitoring was extended until 12 h after seizures being controlled. Genetic polymorphisms in UGT1A6, UGT2B7, CYP2C9, and CYP2C19 were analyzed in association with daily VPA plasma concentrations, adjusted dosages, and occurrence of seizures.

**Results:**

Among the 395 recruited patients, eighty-three (21%) had early post-traumatic seizure, of which 30 (36.14%) were non-convulsive. Most seizures were first detected on day 1 (34.94%) and day 2 (46.99%) after injury. Patients with seizure had longer ICU length of stay and relatively lower VPA plasma concentrations. Patients with UGT1A6_19T > G/541A > G/552A > C double heterozygosities or CYP2C9 extensive metabolizers (EMs) initially had lower adjusted VPA plasma concentrations (power >0.99) and accordingly require higher VPA dosages during later time of treatment (power >0.99). The odds ratio indicated a higher risk of early post-traumatic seizure occurrence in male patients (OR 1.96, 95% CI 1.01-3.81, p = 0.043), age over 65 (OR 2.13, 95% CI 1.01-4.48), and with UGT1A6_19T > G/541A > G/552A > C double heterozygosities (OR 2.38, 95% CI 1.11-5.10, p = 0.02), though the power of the difference was between 0.54 to 0.61.

**Discussion:**

Due to limited facility, the actual frequency of non-convulsive seizures is suspected to be higher than identified. There has been discrepancy regarding to genetic polymorphisms and VPA metab olism between this study and some previous reports. This could be related to confounders such as sample size, race, and patient age. Another limitation is that the case numbers of certain genotypes are limited in this study.

**Conclusions:**

Continuous EEG monitoring is necessary to detect both convulsive and non-convulsive early post-traumatic seizures in severe TBI patients. UGT1A6/CYP2C9 polymorphisms have influence on VPA metabolism. UGT1A6_19T > G/541A > G/552A > C double heterozygositie is associated with occurrence of early post-traumatic seizures in addition to patients’ age and gender. Further investigations with larger sample size are required to confirm the difference.

**Trial registration:**

Retrospectively registered with Chinese Clinical Trail Registry on 1^st^ Jan 2016 (ChiCTR-OPC-16007687)

**Electronic supplementary material:**

The online version of this article (doi:10.1186/s13049-017-0382-0) contains supplementary material, which is available to authorized users.

## Background

Seizure is one of the major complications of traumatic brain injury (TBI). Previous studies have indicated that post-traumatic seizures occur in approximately 16.7-28% of patients with blunt TBI [1–5]. More importantly, nearly half of these were found to be non-convulsive that could only be detected by continuous electroencephalograph (EEG) monitoring [2, 6]. Seizures after TBI may lead to elevated intracranial pressure, cerebral metabolic distress, additional brain damage, and eventually a worse outcome [7, 8]. Compared to convulsive seizures, non-convulsive seizures (NCS) may have greater impact on patients’ outcome as they are not easy to detect.

The current guidelines for the management of severe TBI suggest prophylactic anti-seizure therapy to decrease the incidence of early posttraumatic seizures (within 7 days after injury) [9]. Valproic acid (VPA) is a common anticonvulsant. It has been widely applied in neurointensive care units (NICU), although there is significant inter-individual variability in VPA plasma concentrations that requires monitoring for dose adjustment [10]. Previous studies have indicated that the metabolism of VPA involves three major biotransformation pathways, which are conjugation with glucuronic acid, mitochrondrial β-oxidation, and CYP-catalyzed terminal desaturation and hydroxylation [11]. Among these pathways, the most predominant one is glucuronidation conjugation, mainly mediated by UGT1A6 and UGT2B7 [12–14]. CYP-catalyzed metabolism of VPA accounting for approximately 10-20% of the administrated dose [15–17]. It has been demonstrated that the above genes encoding VPA metabolizing enzymes are highly polymorphic, with promoter single nucleotide polymorphisms (SNPs), which have significant impacts on drug metabolisms including VPA [14, 18–21]. However, the reported contributions of genetic variations on VPA disposition were highly inconsistent, and their influences on early post-traumatic seizures and NCSs have not been explored. The aim of this study is to investigate the incidence of early post-traumatic seizures and the influence of genetic polymorphisms on VPA serum concentrations in a cohort of Chinese patients with severe TBI.

## Methods

### Patient population

From June 2012 to June 2016, a prospective cohort case control study was conducted in Shanghai Huashan Hospital. Adult patients (age ≥ 16) with severe blunt and isolated TBI (Glasgow Coma Score, GCS ≤ 8) were recruited. TBI was defined by a head Abbreviated Injury Scale (AIS) ≥2 [22] or any intracranial hematoma (e.g. cerebral contusion; subarachnoid, subdural or epidural hemorrhages) seen on head CT scans [23–25]. Patients in extremis, who were deemed unlikely to survive three days, were excluded. Exclusion criteria also included: an extracranial AIS ≥ 2; penetrating trauma, body weight > 90kg or <40kg; pregnancy; patients with pre-existing seizures; a history of cirrhosis or renal failure, and allergies to VPA. Since VPA may interact with other drugs’ metabolism, patients who required to take the following medicine within 7 days after injury were also excluded: aspirin, carbapenem antibiotics, cimetidine, erythromycin, felbamate, mefloquine, oral contraceptives, and rifampin.

### Clinical management

TBI patients arriving in the Emergency were consecutively screened by emergency physicians and then by neurosurgeons on duty. They both had received proper training for AIS and GCS assessment. Patients with severe TBI were admitted into NICU directly or after surgical procedures. Operational and conservative treatment were performed under the guidelines of severe TBI management [26]. Patients’ ICU length of stay (ICULOS), hospital length of stay (HLOS), and mortality were recorded at discharge. The clinical outcome was categorized after 3 months follow-up according to Glasgow Outcome Scales (GOS). GOS score of three or less was considered unfavorable.

### EEG monitoring

Continuous EEG monitoring was performed using a bedside equipment (Nemus 2, EB Neuro SpA, Firenze, Italy) as soon as recruited patients being admitted into NICU. Due to limited availability of bedside equipment, EEG monitoring was performed for approximately 4 h for each patient per day from day 1 to day 7 after injury. For patients with suspicious convulsive or non-convulsive seizures, Head CT scans and blood tests were performed in the first instance.to rule out expanding of intracranial lesions or disturbance of internal environment. In the mean time, continuous EEG monitoring was extended for these patients until 12 h after seizures being controlled or ruled out. EEG was recorded digitally using 21 electrodes placed according to the International 10–20 System. Recordings were reviewed daily by two electrophysiologists jointly. Digital videos were screened when episodes of electrographic seizures were detected. Definitions for seizures are consistent with previous publications [3]. Convulsive seizures were considered when the following clinical episodes were described: “generalized tonic-clonic seizures,” “grand mal seizures,” “convulsions,” “rhythmic jerking,” “rhythmic twitching,” or similar descriptions. If EEG confirmed seizures without the above symptoms, such seizures were considered non-convulsive.

### Anti-seizure treatment

VPA was applied as prophylactic anti-seizure therapy during the first 7 days after injury. In order to rapidly achieve stable a dosage regimens, 0.4g of VPA was injected after NICU admission followed by continuous intravenous infusion with a starting dosage of 1.6g per day. Initial VPA dosing was decided referring to established unit protocols and early publications, particularly studies based on Chinese populations [27–30]. Peripheral blood samples were collected from patients every morning and plasma VPA concentrations were quantified using the fluorescence polarization immunoassay. Since it usually took two working days to achieve plasma VPA concentration data, the adjustment of VPA dosages often started on day 3 or day 4 after recruitment, by escalating or decreasing 5–10 mg/kg/day [31] to reach the target concentrations and not to exceed the maximum tolerated dosage. The targeting plasma VPA concentrations was 50 ~ 100μg/ml and the maximum VPA dosage was 3,000mg/day or 40mg/kg/day. Adjusted serum VPA concentration (μg/ml per mg/kg) = Serum VPA concentration (μg/ml)/[VPA daily dose (mg per day)/ weight (kg)]. Midazolam was administered intravenously as additional first-line treatment in cases of convulsive or non-convulsive seizure that could not been controlled by VPA infusion alone, followed by previous studies and guidelines that were summarized elsewhere [32]. Other antiepileptic drugs such as levetiracetam were not applied in this study during the first week of treatment. However, they were considered to be applied either alone or in combination to treat post-traumatic epilepsy (occurred later than 7 days after TBI) if it could not be controlled by VPA.

### Genotyping

Peripheral blood samples for genotyping was collected from each TBI patient using a QIAamp® DNA Mini Kit (QIAGEN, Hilden, Germany). Polymorphism was genotyped by polymerase chain reaction-restriction fragment length polymorphism (PCR-RFLP) for UGT1A6_19t > G/541A > G/552A > C, UGT2B7_ 802C > T/211G > T, CYP2C9*2_430C > T, CYP2C9*3_1075A > C, CYP2C19*2_681G > A, and CYP2C19*3_636G > A, performed by the Clinical Laboratory Department using protocols described elsewhere [19, 33, 34]. The CYP2C19 genotypes were classified into homozygous extensive metabolizers (EMs, *1/*1), intermediate metabolizers (IMs, *1/*2 or *1/*3), and poor metabolizers (PMs, *2/*2, *3/*3, or *2/*3). Similarly, the CYP2C9 genotype were classified as EMs (*1/*1), IMs (*1/*2 or *1/*3), and PMs (*2/*2, *3/*3, or *2/*3).

### Statistical analysis

Statistical analysis was carried out using STATA 13 (StataCorp LP, College Station, TX, USA) and Excel 2013® (Microsoft Corp. Redmond WA) software. The quantitative data were expressed as the mean ± standard deviation (SD). Shapiro-Wilk W test and F test were performed for normality and homogeneity of variance. Independent samples *t*-test, satterthwaite t’ test, Wilcoxon rank-sum test, or one-way analysis of variance (ANOVA) was used for the quantitative data of independent groups. The chi-square test was used for qualitative data. Fisher's exact test was used in the analysis of contingency tables where sample sizes are small (*n* < 40). Differences were considered statistically significant when *p* < 0.05. Power calculations were performed when significant differences were detected for both quantitative and qualitative data, using 2-samples, 2 sides, 2-means or 2-proportions test (alpha = 0.05).

## Results

### Patient demographics

During the period of our study, 1075 patients with head injuries were admitted in our institution, of which 395 (303 males and 92 females) met the inclusion criteria. The demographic data is summarized in Table 1. CT scan showed subarachnoid hemorrhage (SAH) in 232 (58.73%) patients; 146 (36.66%) patients had subdural hemorrhage (SDH) and 205 (51.90%) had intracerebral parenchyma hemorrhage. The frequencies of epidural hemorrhage (EDH), diffuse axonal injury (DAI) and intraventricular hemorrhage (IVH) were 19.0%, 11.1% and 9.1%, respectively. Skull fracture was observed in 266 (67.34%) cases. The mortality of recruited sever TBI patients was 13.67% at discharge and 15.19% after 3 months follow-up.

**Table 1:** Patient demographics and TBI measurements

Demographic characteristics	Value
Number of patients	395
Male: female ratio	303:92
Age (years)	46.72 ± 18.23
Bodyweight (kg)	72.35 ± 11.61
Cause of injury	
Traffic accidents	269 (68.10%)
Fall	63 (15.95%)
Violent assaults	32 (8.10%)
Others	31 (7.85%)
Type of brian injury^a^	
SAH	232 (58.73%)
SDH	146 (36.66%)
Parenchyma hemorrhage	205 (51.90%)
EDH	75 (18.98%)
DAI	44 (11.14%)
IVH	36 (9.11%)
Skull fracture	266 (67.34%)
Head AIS	
2	87 (22.03%)
3	131 (33.16%)
4	116 (29.37%)
5	61 (15.44%)
6	0 (0%)
GCS	
7-8	190 (48.10%)
5-6	156 (39.49%)
3-4	49 (12.41%)
Alanine transaminase (ALT)	14.62 ± 8.21
Aspartate aminotransferase (AST)	21.44 ± 7.83
Creatinine (Cr)	38.75 ± 11.40
Mortality	60/395 (15.19%)

^a^Multi-type injuries (e.g. skull fracture with EDH) were identified in 41.26% TBI patients

### Incidence of early post-traumatic seizures

**Fig. 1:**
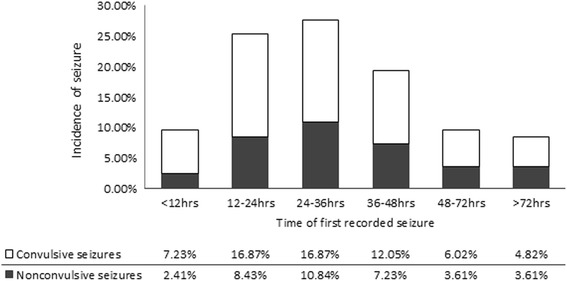
Time and proportions of first seizures within 7 days after severe TBI

Early post-traumatic seizures were detected in 83 (21.01%) of the 395 TBI patients. Over one third of the recorded seizures were non-convulsive (*n* = 30, 36.14%). Fifty-three patients had convulsive seizures, and sixteen of them had both convulsive seizures and NCS. Twenty-nine (34.94%) patients had their first recorded seizure within 24 h after injury. In 39 (46.99%) cases, the first seizure was recorded on day2. Eight (9.64%) cases had first seizure attack on day3, and 6 (8.43%) patients had their first detected seizures later than 72 h of monitoring (Fig. 1). The occurrence of early post-traumatic seizures was associated with patient outcome. As Table 2 indicates, patients who had seizure had longer ICULOS than those without seizure, although their longer HLOS, mortality, and long-term GOS showed no significant difference.

**Table 2:** Characters of TBI patients with/without early post-traumatic seizure

	With seizure (*n* = 83)	Without seizure (*n* = 312)	*p*-value
Head AIS (n, %)			
2	18, 21.69	69, 22.11	0.93
3	25, 30.12	106, 33.97	0.51
4	26, 31.23	90, 28.85	0.66
5	14, 16.87	47, 15.06	0.69
GCS (n, %)			
7-8	41, 49.40	149, 47.76	0.79
5-6	33, 39.76	123, 39.42	0.96
3-4	9, 10.84	40, 12.82	0.63
VPA concentrations (ug/ml): mean ± sd, median			
Day1	59.12 ± 24.18, 59.0	63.96 ± 16.60, 62.5	*0.03*
Day2	61.53 ± 18.23, 62.0	65.92 ± 15.37, 62.0	*0.03*
Day3	63.63 ± 19.03, 63.0	69.05 ± 26.30, 71.5	0.08
Day4	65.56 ± 16.26, 66.0	68.24 ± 24.18, 70.0	0.35
Day5	68.14 ± 24.18, 69.0	70.05 + 23.26, 68.0	0.53
Day6	73.22 ± 29.13, 70.0	75.02 ± 27.04, 73.5	0.61
Day7	69.92 ± 26.04, 72.0	71.35 + 21.57, 72.5	0.62
Weight (kg): mean ± sd, median	73.27 ± 12.71, 72.0	72.12 ± 10.95, 70.5	0.41
ICULOS (days): mean ± sd, median	19.25 ± 6.52, 18.0	15.31 ± 7.10, 16.0	*<0.01*
HLOS (days): mean ± sd, median	20.32 ± 7.35, 21.0	19.01 ± 7.78, 19.0	0.17
Mortality at discharge (n, %)	11,13.25	43, 13.78	0.90
Mortality at 3 months after injury (n, %)	13, 15.66	47, 15.06	0.97
GOS at 3 months after injury			
1-3 (n, %)	72, 86.74	256, 82.05	0.31
4-5 (n, %)	11, 13.25	56, 17.94	0.31

Differences were considered statistically significant when *p*<0.05.

*sd* standard deviation

### Genetic polymorphisms in association with VPA concentrations, dosages and early post-traumatic seizures

Interestingly, we found patients who had early post-traumatic seizure attack had relative lower plasma VPA concentrations compared to those who did not have seizure during the first two days after injury. To explore whether such difference is related to genetic mutations, we analyzed polymorphisms of UGT1A6, UGT2B7, CYP2C9 and CYP2C19 polymorphisms. The genetic variations and corresponding frequencies are shown in Additional file 1: Table S1. The frequency of each genotype was consistent with the HWE. For UGT1A6, patients with double heterozygosities at nucleotide positions 19T > G/541A > G/552A > C had significantly lower VPA plasma concentrations during day 1-2 after injury than those with wild type or single heterozygosity. Accordingly, these patients required higher VPA daily dosages in the following treatment to increase plasma concentrations. Similarly, patients with CYP2C9 EMs had significantly lower VPA concentrations than those with IMs in the first two days of treatment, and required higher VPA dosages during the later treatment. The VPA concentrations and required dosage of CYP2C9 PMs were similar to those of CYP2C9 IMs, though due to limited case number (*n* = 6) they were not statistically different compared to measurements of CYP2C9 EMs. Patients with the rest analyzed SNPs showed no significant difference in VPA plasma concentrations or requiring dosages. When patients were stratified by gender and age, a significant association was observed between age over 65 and initially higher VPA plasma concentrations. However, such relationship was not found between male and female patients. Patients with UGT1A6_19T > G/541A > G/552A > C double heterozygosities had a statistically higher occurrence rate of early post-traumatic seizure compared to those with wild type. The odds ratio also indicated a higher risk of seizure occurrence in male patients and age over 65. However, the power of the above difference is 0.61, 0.53, and 0.53, respectively, which indicates further investigations with larger sample size are required. Although the proportion of early post-traumatic seizure attacks in patients with CYP2C9 EMs was higher than those with IMs or PMs, the difference was not significant (Additional file 1: Table S1).

## Discussion

Seizure is a common complication in all types of acute brain injuries. Previous studies have revealed that up to 28% of blunt TBI patients may have post-traumatic seizures [1–5]. Continuous EEG monitoring reveals that approximately 10% of patients have nonconvulsive seizures, with 30% presenting in the first three days after injury [2, 3, 35]. It has been recognized that seizure is associated with increased brain edema, excessive metabolic demand, elevated intracranial pressure, and worsened brain atrophy [7, 8, 36]. Such pathologic process should be diagnosed and controlled as early as possible. In this study, even after excluding patients with history of seizures, still 83 (21.01%) of severe TBI patients had seizure during the first week after injury. More importantly, thirty out of 83 (36.14%) were identified as NCSs that could only be detected by continuous EEG monitoring. These findings were close to previous publications [3–5]. In fact, we suspect that the actual frequency of NCSs could be higher than we identified. Some patients with subclinical seizures may not have been detected, as we only performed approximate 4 h continuous EEG monitoring for each patient due to limited equipment and conducting other examinations or therapies. The majority (67 of 83, 80.72%) cases had their seizure attacks within 48 h of monitoring. The peak incidence appeared in 12-24 h (25.30%) and 24-36 h (27.71%) after injury. Over 9% of patients did not have their first seizure until 48 h after injury, and 8.43% seizures were first detected at 72 h or later. This suggests that prolonged continues EEG monitoring for sever TBI patients is necessary.

Considering the high incidence, one may reasonably expect worse outcomes in patients with post-traumatic seizures. In this study, the impact of early post-traumatic seizures on clinical outcomes were reflected by prolonged ICULOS. This could be explained that patients with post-traumatic seizure requires longer ICU stay for EEG monitoring, extended periods for anti-seizure therapies, and other intensive care for complications. However, the HLOS showed no significant difference between groups. A possible explanation is that severe TBI patients were all transferred to rehabilitation centers for further therapies once their situations were stable. The mortalities and 3-month GOS also showed no significant difference between the two groups. This could due to the fact that the prognosis of sever TBI is influenced by many other factors, such as increased intracranial pressure, hospital acquired pneumonia, coagulation abnormalities, rehabilitation therapies, etc. To better determine the associations between seizures and patient outcomes, it would require further investigations with more sophisticated designs to screen each confounder.

The new generation of anti-epileptic medications such as levetiracetam, topiramate, lamotrigine appears to be increasing in use for seizure prophylaxis. However, to date there have been insufficient data to support their superiority or inferiority compared to other anticonvulsants [9]. For patients with severe TBI and in coma, VPA for injection has the advantage of being easy to administrate and affordable. For example, VPA in East China is approximately $0.02 per 0.2g, $0.2 per 0.5g of sustained release tablets, and $12 per 0.4g of injection. Levetiracetam pills, in the contrast, are approximately $1.4 per 0.5g. Levetiracetam injections are not available in China. Topiramate and Lamotrigine pills are both around $0.7 per 50mg. Therefore, in many countries, particularly for patients in less developed areas, VPA it is still the first line antiepileptic drug. As mentioned earlier, the antiepileptic effects of VPA requires stable plasma concentrations, which are associated with genetic polymorphisms affecting VPA pharmacokinetics and pharmacodynamics. Compared to previous studies, there were no significant differences in the frequencies of the UGT1A6, UGT2B7, CYP2C9, CYP2C19 genotypes between our observations and other reported Chinese populations [29, 37–39]. During the first two days after injury, when plasma concentrations were initially not available, all patients received similar dosages of VPA injection in our study. Under this circumstance, patients with UGT1A6 double heterozygosities at nucleotide positions 19T > G, 541A > G and 552A > C had lower adjusted plasma VPA concentrations compared to those with wild type or single heterozygosity. Accordingly, these patients received higher dose of VPA injection to achieve targeting plasma concentrations during later days of monitoring. Similarly, patients with wild type CYP2C9 (EMs) were associated with lower adjusted VPA plasma concentrations during the first 2 days after injury and higher VPA dosages at later time. Among the other studied SNPs, the adjusted plasma VPA concentrations and VPA dosages showed no significant difference. These results suggested that UGT1A6 double heterozygosities had higher enzyme activity and intrinsic clearance. The robust VPA terminal desaturation and hydroxylation in patients with CYP2C9 EMs may also be responsible for decreased adjusted plasma VPA concentrations.

Previously, there have been studies investigating SNPs in genes relating to VPA metabolism and disposition either alone or in combination. However, the results were not entirely consistent. For example, our findings on UGT1A6 SNPs were supported by two recent publications showing that homozygous carriers of the variant UGT1A6 19T > G, 541A > G and 552A > C allele have relatively low VPA concentrations [39, 40]. However, similar results were not achieved in other studies [41]. In addition, while there have been studies demonstrating the impact of CYP2C9 and/or CYP2C19 polymorphisms on VPA pharmacokinetics [28, 42], other studies claimed no association between the CYP2C9 genotypes and VPA plasma concentrations or incidence of seizure [29, 43, 44]. We suppose such discrepancy lies in the fact that VPA is metabolized by multiple UGTs and CYPs, and the disposition could be related to other factors such as sample size, race, and patient age. In our studies we found patients over 65 years old had higher adjusted VPA plasma concentrations and required less adjusted VPA dosages.

Our study has several limitations. Although we have recruited potential patients for a period of four years, the number of cases with certain genotypes, such as CYP2C9 PMs, were still limited, which led to statistically significant difference in neither VPA concentrations nor VPA dosages. Similarly, although it is identified that male, age over 65, and UGT1A6 double heterozygosities were associate with higher incidence of early post-traumatic seizures, the powers of the difference were less than 0.80. Therefore, further single- or multi-center investigations with larger sample size are required to increase the power and to confirm the difference. Another undeniable fact is that the pathogenesis of seizure and its influence factors are not fully understood. Although VPA is a broad-spectrum antiepileptic drug, reaching the effective blood concentration does not equal to avoidance of seizure attack. New antiepileptic medications have been more popular, yet their pharmacokinetics and relationship to SNPs are mostly complicated and remain unclear. In our study, early post-traumatic seizures were treated using first and second line anti-seizure drugs [32]. We did not change the therapeutic regime after genetic polymorphism analyzing. This is partly due to genetic polymorphism analyzing requires several days in our institution, too long for a window of 7 days of early post-traumatic seizures. However, this would be an attractive issue to address in the future study. In sum, the above questions reinforce the need for further investigations and to promote precision medicine in the area of anti-seizure therapy.

## Conclusions

Continuous EEG monitoring were necessary to detect convulsive and non-convulsive post-traumatic seizures in patients with severe TBI. VPA metabolism were affected by UGT1A6/CYP2C9 mutations. It would be worthwhile to perform UGT/CYP polymorphisms screen and VPA plasma concentration monitoring within 7 days after injury. Male patients, age over 65, and with UGT1A6_19T > G/541A > G/552A > C double heterozygosities had higher risk of early post-traumatic seizures, but further investigations with larger sample size are required to better illustrate the difference.

## Additional file

Additional file 1: Table S1. Genetic polymorphism, gender, and sex in association with VPA concentrations, VPA dosages and early post-traumatic seizures. (DOCX 25 kb)

## Abbreviations

DAI: Diffuse axonal injury
EDH: Epidural hemorrhage
EEG: Electroencephalograph
EMs: Extensive metabolizers
GCS: Glasgow Coma Score
GOS: Glasgow Outcome Scales
HLOS: Hospital length of stay
ICULOS: ICU length of stay
IMs: Intermediate metabolizers
IVH: Intraventricular hemorrhage
NCS: Non-convulsive seizures
NICU: Neurointensive care units
PMs: Poor metabolizers
SAH: Subarachnoid hemorrhage
sd: Standard deviation
SDH: Subdural hemorrhage
SNPs: Single nucleotide polymorphisms
TBI: Traumatic brain injury
VPA: Valproic acid
