# Supplementary material for: Early post-traumatic seizures are associated with valproic acid plasma concentrations and UGT1A6/CYP2C9 genetic polymorphisms in patients with severe traumatic brain injury
Source: Scand J Trauma Resusc Emerg Med. 2017 Aug 25;25:85. doi: 10.1186/s13049-017-0382-0 (PMC5574127; doi:10.1186/s13049-017-0382-0)
Supplement: Additional file 1: Table S1. — Genetic polymorphism, gender, and sex in association with VPA concentrations, VPA dosages and early post-traumatic seizures. (DOCX 25 kb) [file 13049_2017_382_MOESM1_ESM.docx]

**Supplementary Table 1. Genetic polymorphism, gender, and sex in association with VPA concentrations, VPA dosages and early post-traumatic seizures**

|  |  |  | Adjusted VPA concentration (ug/ml per mg/kg) | | | |  | VPA dosages (mg/kg per day) | | | |  | Early seizure | | |
| --- | --- | --- | --- | --- | --- | --- | --- | --- | --- | --- | --- | --- | --- | --- | --- |
| Categories | n | % | Day1-2 | p_1_^†^, p_2_^‡^ | Day3-7 | p_1_, p_2_ |  | Day1-2 | p_1_, p_2_ | Day3-7 | p_1_, p_2_ |  | n,(%) | OR (95% CI) | p_3_^§^ |
| UGT1A6_19T>G/541A>G/552A>C ^II^ |  |  |  |  |  |  |  |  |  |  |  |  |  |  |  |
| Wild type | 201 | 50.89 | 4.31±1.76 | /,/ | 3.98±1.80 | /,/ |  | 17.54±2.49 | /,/ | 17.54±7.49 | /,/ |  | 36(17.91) | 1 | / |
| Single heterozygosity | 156 | 39.49 | 4.36±1.85 | 0.79,/ | 4.25±1.37 | 0.12,/ |  | 17.27±2.01 | 0.27,/ | 17.27±8.01 | 0.74,/ |  | 34 (21.79) | 1.28（0.76-2.16） | 0.36 |
| Double heterozygosities | 38 | 9.62 | 2.58±1.92 | **<0.01^¶^,<0.01^¶^** | 4.17±1.37 | 0.64,0.74 |  | 17.58±2.15 | 0.92,0.40 | 24.58±5.23 | **<0.01**^¶^**,<0.01**^¶^ |  | 13 (34.21) | 2.38（1.11-5.10） | **0.02**^#^ |
| UGT2B7*71S_211G>T |  |  |  |  |  |  |  |  |  |  |  |  |  |  |  |
| Wild type | 256 | 64.81 | 4.36±1.52 | /,/ | 4.23±1.43 | /,/ |  | 17.06±2.20 | /,/ | 17.23±3.14 | /,/ |  | 54(21.09) | 1 | / |
| Heterozygosity | 121 | 30.63 | 4.18±1.91 | 0.32,/ | 4.19±1.22 | 0.32,/ |  | 17.73±2.13 | 0.056,/ | 17.37±3.65 | 0.70,/ |  | 26(21.49) | 1.03 (0.60-1.74) | 0.93 |
| Homozygosity | 17 | 4.30 | 3.97±1.87 | 0.31,0.67 | 4.13±1.62 | 0.31,0.51 |  | 17.50±1.91 | 0.42,0.67 | 17.82±2.24 | 0.44,0.62 |  | 3(17.65) | 1.15 (0.36-3.67) | 0.81 |
| UGT2B7*2_802G>T |  |  |  |  |  |  |  |  |  |  |  |  |  |  |  |
| Wild type | 201 | 50.89 | 3.99±1.03 | /,/ | 4.21±1.56 | /,/ |  | 17.31±2.31 | /,/ | 17.54±2.67 | /,/ |  | 41 (20.40) | 1 | / |
| Heterozygosity | 149 | 37.72 | 4.11±1.25 | 0.32,/ | 4.07±1.47 | 0.40,/ |  | 17.42±3.14 | 0.70,/ | 18.09±3.52 | 0.097,/ |  | 33(22.15) | 1.11 (0.66-1.86) | 0.69 |
| Homozygosity | 45 | 11.39 | 4.18±1.23 | 0.28,0.54 | 3.97±1.41 | 0.34,0.69 |  | 17.49±2.26 | 0.61,0.89 | 17.03±3.24 | 0.27, 0.073 |  | 9(20.00) | 0.98 (0.44-2.19) | 0.95 |
| CYP2C9*2_430C>T)/CYP2C9*3_1075A>C |  |  |  |  |  |  |  |  |  |  |  |  |  |  |  |
| EMs *1/*1 （*2CC, *3AA) | 285 | 72.15 | 3.23±1.86 | /,/ | 4.04±1.50 | /,/ |  | 17.74±2.70 | /,/ | 19.35±3.79 | /,/ |  | 55(19.30) | 1 | / |
| IMs *1/*2 or *1/*3 (*2CT/*3AA or *2CC/*3AC) | 104 | 26.33 | 4.24±2.01 | **<0.01**^¶^**,/** | 4.15±1.32 | 0.51,/ |  | 18.07±1.97 | 0.25,/ | 17.25±3.26 | **<0.01**^¶^,/ |  | 26(25.00) | 1.39(0.81-2.37) | 0.22 |
| PMs *2/*2,*3/*3,*2/*3 (*2TT/*3AA,*2CT/*3AC,*2CC/*3CC) | 6 | 1.52 | 4.56±1.91 | 0.08**,**0.07 | 3.97±1.41 | 0.91,0.75 |  | 17.07±1.93 | 0.55, 0.23 | 16.84±2.64 | 0.11**,** 0.76 |  | 2(33.33) | 2.09(0.37-11.70) | 0.39 |
| CYP2C19*2_636G>A)/CYP2C19*3_681G>A) |  |  |  |  |  |  |  |  |  |  |  |  |  |  |  |
| EMs *1/*1 （*2GG, *3GG) | 171 | 43.29 | 4.04±1.34 | /,/ | 4.15±1.30 | /,/ |  | 17.14±2.31 | /,/ | 17.46±2.84 | /,/ |  | 35(20.47) | 1 | / |
| IMs *1/*2 or *1/*3 (*2GG/*3GA or *2GA/*3GG) | 173 | 43.80 | 3.93±1.96 | 0.544,/ | 3.92±1.26 | 0.097,/ |  | 17.49±2.14 | 0.14,/ | 18.02±3.12 | 0.82,/ |  | 35(20.23) | 1.01(0.60-1.72) | 0.95 |
| PMs *2/*2,*3/*3,*2/*3 (*2GG/*3AA,*2AA/*3GG,*2GA/*3GA) | 51 | 12.91 | 4.35±1.91 | 0.19,0.18 | 4.03±1.41 | 0.57,0.59 |  | 17.11±2.17 | 0.93,0.26 | 17.96±3.28 | 0.29， 0.91, |  | 13(25.49) | 1.35(0.65-2.80) | 0.42 |
| Gender |  |  |  |  |  |  |  |  |  |  |  |  |  |  |  |
| Female | 92 | 23.29 | 4.25±2.08 | /,/ | 4.15±1.30 | /,/ |  | 17.26±3.04 | /,/ | 17.69±3.18 | /,/ |  | 12(13.04） | 1 | / |
| Male | 303 | 76.71 | 4.14±1.98 | 0.64 | 4.03±1.41 | 0.60 |  | 17.72±2.32 | 0.18 | 17.95±3.22 | 0.50 |  | 69（22.77） | 1.96(1.01-3.81) | **0.043**^#^ |
| Age |  |  |  |  |  |  |  |  |  |  |  |  |  |  |  |
| >65 years old | 82 | 20.76 | 4.32±2.14 | /,/ | 4.15±1.32 | /,/ |  | 17.47±2.98 | /,/ | 16.58±3.63 | /,/ |  | 9（10.98） | 1 | / |
| ≤65 years old | 313 | 79.24 | 3.26±1.79 | **<0.01**^¶^ | 4.07±1.47 | 0.67 |  | 17.28±2.31 | 0.54 | 17.86±3.31 | **<0.01**^¶^ |  | 65（20.77） | 2.13(1.01-4.48) | **0.043**^#^ |

† The p_1_ values indicates significance of difference between measurements from subjects with wild type genes *vs.* single heterozygosity, or IMs *vs.* EMs.

‡The p_2_ values indicates significance of difference between measurements from subjects with doulbe heterozygosities *vs.* single heterozygosity, homozygosities vs. heterozygosities, or PMs *vs.* IMs.

§The p_3_ values indicates significance of difference in occurrence of early post-traumatic seizures between subjects with wild type genes and mutations.

II Wild type in UGT1A6 gene is defined as *1/*1; Single heterozygosity includes *1/*2, *1/*3, and *1/*4; double heterozygosities include *2/*2, *2/*4; *2 is defined as 19G/541G/552C; *3 is defined as 19G/541A/552A; *4 is defined as 19G/541A/552C .

¶ Power>0.80.

# Power <0.80.

OR: odds ratio.
